# Effects of Telerehabilitation Platforms on Quality of Life in People with Multiple Sclerosis: A Systematic Review of Randomized Clinical Trials

**DOI:** 10.3390/neurosci6040103

**Published:** 2025-10-13

**Authors:** Alejandro Herrera-Rojas, Andrés Moreno-Molina, Elena García-García, Naiara Molina-Rodríguez, Roberto Cano-de-la-Cuerda

**Affiliations:** 1 Department of Physical Therapy, Occupational Therapy, Physical Medicine and Rehabilitation, Faculty of Health Sciences, Rey Juan Carlos University, 28922 Alcorcón, Spain; 2 Grupo de Investigación de Neurociencias Aplicadas a la Rehabilitación (GINARE), 28923 Alcorcón, Spain; 3 Departamento de Fisioterapia, Centro Superior de Estudios Universitarios La Salle, Universidad Autónoma de Madrid, Aravaca, 28049 Madrid, Spain; 4 Physical Therapy Service, Primary Care, Servicio Madrileño de Salud (SERMAS), 28046 Madrid, Spain; 5 Instituto de Salud Integral EZMA, 28944 Madrid, Spain

**Keywords:** information technologies, multiple sclerosis, physical therapy, quality of life, rehabilitation, telerehabilitation

## Abstract

Introduction: Multiple sclerosis (MS) is a chronic neurodegenerative disease that entails high costs, progressive disability, and reduced quality of life (QoL). Telerehabilitation (TR), supported by new technologies, is emerging as an alternative or complement to in-person rehabilitation, potentially lowering socioeconomic impact and improving QoL. Aim: The objective of this study was to evaluate the effect of TR on the QoL of people with MS compared with in-person rehabilitation or no intervention. Materials and methods: A systematic review of randomized clinical trials was conducted (March–May 2025) following PRISMA guidelines. Searches were run in the PubMed-Medline, EMBASE, PEDro, Web of Science, and Dialnet databases. Methodological quality was assessed with the CASP scale, risk of bias with the Risk of Bias 2 tool, and evidence level and grade of recommendation with the Oxford Classification. The protocol was registered in PROSPERO (CRD420251110353). Results: Of the 151 articles initially found, 12 RCTs (598 total patients) met the inclusion criteria. Interventions included (a) four studies employing video-controlled exercise (one involving Pilates to improve fitness, another involving exercise to improve fatigue and general health, and two using exercises focused on the pelvic floor muscles); (b) three studies using a monitoring app to improve manual dexterity, symptom control, and increased physical activity; (c) two studies implementing an augmented reality system to treat cognitive deficits and sexual disorders, respectively; (d) one platform with a virtual reality headset for motor and cognitive training; (e) one study focusing on video-controlled motor imagery, along with the use of a pain management app; (f) a final study addressing cognitive training and pain reduction. Studies used eight different scales to assess QoL, finding similar improvements between groups in eight of the trials and statistically significant improvements in favor of TR in four. The included trials were of good methodological quality, with a moderate-to-low risk of bias and good levels of evidence and grades of recommendation. Conclusions: TR was more effective in improving the QoL of people with MS than no intervention, was as effective as in-person treatment in patients with EDSS ≤ 6, and appeared to be more effective than in-person intervention in patients with EDSS between 5.5 and 7.5 in terms of QoL. It may also eliminate some common barriers to accessing such treatments.

## 1. Introduction

Multiple sclerosis (MS) is a chronic neuroinflammatory autoimmune disease that damages the myelin of axons in the central nervous system (CNS) [1]. It is characterized by the presence of focal lesions in the form of demyelinating plaques in the CNS, with varying degrees of inflammation, gliosis, and neurodegeneration, which decrease nerve conduction [2]. Its etiology is currently unknown, and a multifactorial origin is assumed [3]. Multiple sclerosis presents with different clinical courses, most commonly relapsing–remitting (RRMS), secondary progressive (SPMS), or primary progressive (PPMS) [4].

In line with the latest review of McDonald diagnostic criteria and the current international phenotype classification, each course can be further described as active or inactive and with or without progression to capture disease activity and disability accumulation [4].

The clinical manifestations of MS result from alterations in various functions, including sensorimotor, bowel, bladder, sexual, brainstem and optic nerve, cerebellar, and neuropsychiatric functions, among others [5]. Treatments can therefore be divided into two categories [6]: “Disease-Modifying Therapy” and “Symptomatic Treatment.” The first consists of immunosuppressive or immunomodulatory drugs, which seek to alter the course of the disease, while symptomatic treatments, whether pharmacological or physical, address symptoms secondary to neurological damage.

Currently, there is no cure for MS, so rehabilitation treatment aims to enhance functionality and manage the observed symptoms. Due to the nature of the disease and the multitude of symptoms and signs exhibited by these patients, a multidisciplinary approach is necessary, involving neurologists, rehabilitation physicians, physiotherapists, occupational therapists, nursing staff, assistants, and social workers, among others, in the individual rehabilitation project [7], to ensure the highest quality of life (QoL) for patients with MS [8,9].

Neurorehabilitation is a process aimed at reducing the disability and social disadvantage suffered by a person as a result of a neurological disease. Its main objective is to reduce the degree of functional impairment [10]. However, as a result of their condition, many patients will experience alterations in their body functions and structures, as well as a decrease in activity and a restriction in participation [8,9,11], which will require medical and social attention, both from their community and from the services that the administration can provide.

The chronicity of MS not only affects the QoL of these patients [12] but also makes access to rehabilitation difficult [13]. Additionally, it increases its costs [14]. For this reason, telerehabilitation (TR), defined as the provision of rehabilitation services through electronic systems based on information and communication technologies (ICTs), is increasingly being considered as a possible implementation tool [15]. One of the fundamental objectives of TR is to provide assistance to the patient in his or her own home, overcoming barriers of time and distance [15]. This approach enables rehabilitation to be extended to a more ecological environment, allowing for better detection of limitations in daily activities and benefits of treatment, while also reducing the costs associated with transportation [15]. A major challenge of TR is developing systems that enable the safe and controlled execution of scheduled tasks, which, according to a recent systematic review, appears to be feasible [16].

TR could have a positive impact on neurological disorders due to environmental enrichment, neuroplasticity enhancement, increased adherence, positive feedback, and the possibility of working on specific tasks in a more enjoyable way and closer to the patient’s daily reality [17]. TR can facilitate the multidisciplinary management of people with MS (PwMS) [18] and provide equal access to geographically remote people and those with physical and economic disadvantages [19]. Furthermore, it has proven to be effective in improving the QoL of patients with different neurological diseases [20]. For this reason, TR is presented as a useful tool in the rehabilitation of PwMS, reducing the costs associated with the disease, the barriers that hinder access to rehabilitation treatments, and being able to have an impact on the QoL of people with MS.

## 2. Aim

The primary purpose of this systematic review is to analyze the effects of TR platforms or programs on the QoL of PwMS compared to conventional presential treatment or no intervention. Secondarily, we aim to study the relationship of the other scales used in the studies with the different domains of the International Classification of Functioning (ICF) (structures/functions, activity, and participation) [11].

## 3. Methods

**Figure 1:**
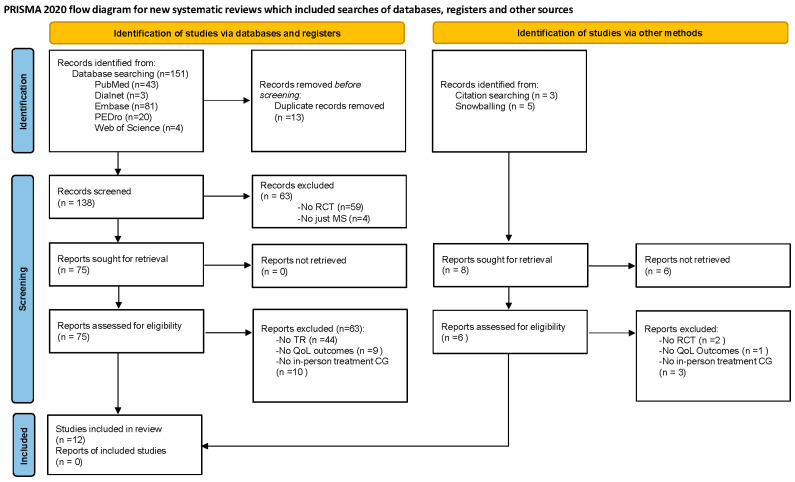
PRISMA flowchart. CG: control group; MS: multiple sclerosis; QoL: quality of life; RCT: randomized controlled Trial; TR: telerehabilitation.

A systematic review was conducted following the Preferred Reporting Items for Systematic Reviews and Meta-Analyses (PRISMA) 2020 statement [21] (Figure 1), posing the following PICO (Population/Intervention/Comparison/Intervention/Outcome(s)): P: people with MS; I: telerehabilitation; C: in-person treatment/or no treatment; O: quality of life.

The present systematic review was registered in PROSPERO with the ID CRD420251110353. The PRISMA checklist for systematic reviews is detailed in Supplementary Material S1.

### 3.1. Search Databases and Strategies

A search of the scientific literature was conducted between March and May 2025 in the international databases PubMed-Medline, EMBASE, PeDro, Web of Science, and Dialnet on improvements in QoL for MS patients who use telerehabilitation strategies during their treatment.

The articles reviewed were written in Spanish and English. The time filter was limited to the last 10 years, and screening was conducted manually by two researchers (A.H.R.) and (E.G.G.), with disagreements resolved by a third researcher (A.M.M.), Zotero software (7.0.26 version) was used to manage, organize, systematize, and integrate bibliographic references.

In the first phase, keywords were identified and transformed into controlled or documentary language terms of the Medical Subject Heading (MeSH) and EMTREE: “Telerehabilitation” [MeSH Terms], “Telemedicine” [MeSH Terms], “Quality of Life” [MeSH Terms], and “Multiple sclerosis” [MeSH Terms]. Subsequently, the advanced search strategies were constructed using Boolean operators (AND, OR and NOT), as well as the search by fields in title and summary, the truncation operator (*), and the filters that were considered most appropriate.

Finally, a review of the literature referenced by the different trials was carried out, and “snowballing” was conducted to analyze the studies included in other systematic reviews on topics related to ours.

The search strategies carried out in each database are presented in Table 1.

### 3.2. Eligibility Criteria

This review considered only studies published in English or Spanish after 2015 and designed as randomized clinical trials (RCTs). All studies that included diseases other than MS, were study protocols, poster or conference presentations, used any design other than an RCT, were published before 2015, or did not include at least one QoL outcome measure were excluded.

Only studies involving people with MS, regardless of type, were included. The inclusion criteria were as follows: (a) people diagnosed with MS; (b) participants randomized to an experimental group undergoing a TR program or to a control group undergoing a face-to-face rehabilitation program or no intervention; (c) studies published after 2015.

### 3.3. Outcomes

The primary outcomes of this review were measures of the QoL in PwMS performed in the included studies.

The remaining scales used in the included trials were also analyzed and taken as secondary outcomes for this review, studying their relationship with the different ICF domains [11].

### 3.4. Results Analysis

After conducting a search and eliminating duplicate documents, a title and abstract analysis was performed to determine which studies initially met the proposed criteria. The full text of the selected studies was then read to make final eliminations and extract relevant information from the included ones. Once this information was collected, it was analyzed and compared with the rest of the articles, and each of the scales used in the trials was assigned to one of the three domains of the ICF classification (body functions and structures, activity, and participation) [11]:

- Body functions and structures: “Anatomical parts and physiological functions of body systems (including psychological functions).”
- Activity: “Performance of a task or action by an individual.”
- Participation: “Act of becoming involved in a life situation.”

Analyses were performed by two researchers (A.H.R. and N.M.R.), and disagreements were resolved by a third researcher (E.G.G.).

### 3.5. Methodological Quality and Risk of Bias Assessment

The methodological quality of the included articles was assessed following the guidelines of the Critical Appraisal Skills Programme (CASP) for RCTs [22]. This scale assesses the internal quality of RCTs using 11 questions, each answering “yes,” “don’t know,” or “no.” A “yes” answer will be given a score of 1, and a 0 will be assigned in the other two cases, giving a maximum score of 11/11. The questions used are as follows: “Is the trial addressing a clearly defined question?”, “Was the allocation of patients to treatment groups random?”, “Was comparability of groups maintained throughout the study?”, “Was management of losses adequate?”, “Was outcome measurement adequate?”, “Was selective reporting of results avoided?”, “What is the effect for each outcome?”, “How precise are the effect estimates?”, “Can this be applied in your local setting or population?”, “Have all outcomes and their clinical importance been considered?”, and “Do the benefits to be obtained justify the risks and costs?”

Furthermore, the Risk of Bias 2 (RoB 2) tool [23] was used to assess the risk of bias for each trial, choosing the “color-blind-friendly” option to generate the results tables in the “robvis” tool [24]. This analysis was based on the Cochrane Handbook of Systematic Reviews of Interventions [25], which identifies five domains:

- Bias arising from the randomization process: This domain refers to whether the randomization process has been carried out and is reflected adequately.
- Bias due to deviations from intended intervention: This section assesses whether both participants and staff followed the intervention as planned, avoiding the loss of imposed blinding.
- Bias due to missing outcome data: These biases arise if, in the event of participant losses during the intervention, an intention-to-treat analysis of the remaining data has not been performed to examine whether they could have altered the results obtained.
- Bias in measurement of the outcome: This section examines whether measurements were made with the correct tools and without being influenced by the participant’s knowledge of the group to which they belong.
- Bias in selection of the reported review: This domain assesses whether the reported results are as planned and whether or not they were selectively displayed based on their statistical significance or other factors.

Lastly, the Oxford Classification [26] was used to define the level of evidence and grade of recommendation for the different trials analyzed. This classification proposes different levels of evidence based on the methodological design used and the quality of the trial, in addition to classifying the recommendation of the proposed intervention according to its results and risk–benefit balance.

## 4. Results

Of the 151 studies initially identified, 12 met the criteria proposed to be part of the review, and 139 were excluded. The results of the searches in the different databases are detailed in the flowchart (Figure 1).

### 4.1. Summary of the Obtained Results

Characteristics of the included articles are summarized in Table 2. The 12 articles included in the review [27,28,29,30,31,32,33,34,35,36,37,38] collected a total sample of 598 patients receiving different TR interventions, with scores on the “Expanded Disability Status Scale” (EDSS) [39] between 0 and 7.5, noting that only 4 studies included patients with EDSS between 6 and 7 [27,30,33,37], levels from which technical aids for walking are necessary [39]. Four of them randomized patients using different computer software [28,29,31,38], three used block randomization [30,33,36], one used a coin toss [35], another one used closed envelopes [34], another trial used the minimization method [32], and two authors did not report the randomization method [27,37]. In terms of the types of MS present in the sample, six studies did not provide data [27,29,32,34,36,38], one only used patients with RRMS [35], another included 87% RRMS and 13% of unspecified types [31], one included RRMS and PPMS [28], two included RRMS and SPMS [30,33], and one included PPMS and SPMS [37]. These trials included patient samples ranging from 30 [34] to 86 participants [35], with a mean participation of 49.8 ± 17.9 subjects.

Regarding the proposed interventions, the following were found: (a) three studies used mobile apps (van Beeck et al. for training manual dexterity [28], Üstundag et al. for controlling disease symptoms [35], and Nasseri et al. for increasing physical activity [37]); (b) one trial used a virtual reality device with offline monitoring by the supervisor to train motor and cognitive skills [29]; (c) two studies conducted by Maggio et al. used the BTS-Nirvana system (one to treat cognitive and motor deficits [30] and another for improvement in the sexual sphere [33]); (d) four carried out exercise interventions (one used Pilates to improve physical fitness [32]; three used different exercise programs to improve pelvic floor functions [31,36], fatigue, general health status, and the ability to perform activities of daily living [38]); and (e) one used motor imagery via an app and video call to monitor patients’ pain [34]; (f) a final trial, conducted by Jeong et al., did not provide information on the TR system implemented to improve QoL, cognitive function, and pain [27].

Regarding the number and timing of sessions, the protocols used interventions that lasted between 4 [28] and 12 weeks [31,35,37,38], with between 1 [36] and 5 weekly sessions [28,29]. The minimum number was 8 sessions (1 weekly for 8 weeks of intervention) [36] and the maximum was 30 (5 weekly, 6 weeks of intervention) [29], with durations between 20 min [36] and 60 [32]. On the other hand, only two trials [28,34] offered a long-term evaluation after 4 [34] and 12 weeks after the end of the trial [28], finding, respectively, decreases [34] and maintenance [28] in the effects of the intervention.

The program supervisors were physical therapists in 9 of the 12 studies [27,29,30,31,32,33,34,36,38]. In one study, supervision was provided by a neurologist [37], in another, by a team consisting of a neurologist, a physiotherapist, and a nurse [35], and in a final article, the supervisor’s profession was not indicated [28].

The studies employed a total of 82 different scales, with 62 used only once and 20 being repeated in different trials. Regarding scales referring to “body structures and functions,” [11] a total of 18 were found, with the most commonly used being the “Fatigue Severity Scale” (FSS), which was used in three articles [29,32,38]. This scale showed results in favor of the TR group in the study by Eldemir et al. [32] compared to the waiting list. It showed results in favor of the in-person control group in the article by Tarakci et al. [38], and both groups showed improvements without statistical differences in the trial conducted by Pagliari et al. [29], with a control group that received conventional in-person treatment. “Activity” [11] was the ICF area that received the most attention, with a total of 27 scales used to measure it, and the “Nine Hole Peg Test” (NHPT), which was repeated three times, standing out [28,29,35]. In this scale, van Beeck et al. [28] showed statistically significant improvement in favor of the TR group compared to the control group, which performed upper limb strength training, and Pagliari et al. [29] and Nasseri et al. [37] found improvements in both groups, without showing significant differences between them, compared to usual care [29] and the delivery of an informative pamphlet [37], respectively.

Lastly, “participation” [11] was the least addressed area, with 18 scales used, and a total of 8 scales analyzing QoL in PwMS. Despite this, the most frequently used scale in the included trials was the “Multiple Sclerosis Quality of Life-54” (MSQoL-54), used in six studies [27,29,30,32,33,35]. Of these studies, those conducted by Pagliari et al. [29], Maggio et al. 2022 [30], Eldemir et al. [31], and Üstundag et al. [35] showed significant improvements in favor of the TR group compared to conventional rehabilitation [29,30,35] and waiting lists [32], while Jeong et al. [27] and Maggio et al. 2024 [33] showed improvements, but without reaching statistical significance. On the other hand, five studies [27,29,30,32,33] did not provide an analysis of the statistical significance of the intergroup differences, although all results seemed to show a benefit in favor of the group that received TR. Only Üstundag et al. [35] offered this analysis, obtaining significantly greater improvements in the MSQoL-54 in the TR group compared to the group that received standard care. The rest of the scales used for QoL measurements were used only once in the included studies and were as follows: “Multiple Sclerosis Impact Scale-29” (MSIS-29) [31], “Kings Health Questionnaire” (KHQ) [36], “Hamburg Quality of Life Questionnaire Multiple Sclerosis” (HAQUAMS) [37], and “Quality of Life Scale” (QoLS) [38]. In the studies conducted by Yavas et al. [31] and Nasseri et al. [37], both study groups improved without significant differences between them, despite the fact that the control groups did not receive any intervention. Bulbul et al. [36] received lifestyle advice, and Tarakci et al. [38] performed supervised in-person exercise. The “Euro Quality of Life” (EuroQoL) [31] and “Multiple Sclerosis Quality of Life” (MusiQoL) [34] showed similar improvements between the TR and in-person intervention groups [31,34] but were significantly greater in the TR groups compared to the untreated control group [31]. Likewise, the specific quality of life scale for urinary function, the “International Consultation of Incontinence Questionnaire-Short Form-Quality of Life” (ICIQ-UI-SF-QoL) was used by Yavas et al. [31], showing greater improvements in the TR group compared to the no-intervention control group.

The classification of all scales used according to the ICF domains, primary ICF codes, and mapping notes are detailed in Supplementary Material S2, and a detailed description of the QoL scales, with particular attention to their psychometric properties, are provided in Supplementary Material S3.

### 4.2. Methodological Quality and Risk of Bias

After analyzing the different sections of the CASP guideline [22], an average internal quality score of 8 ± 1.27 points was obtained, indicating a moderate–high quality level. The studies with the highest scores were those conducted by Karakas [34] and Nasseri [37], with 10 points, and the lowest, by Jeong [27], with 6. The scores for each study are detailed in Table 3.

**Figure 2:**
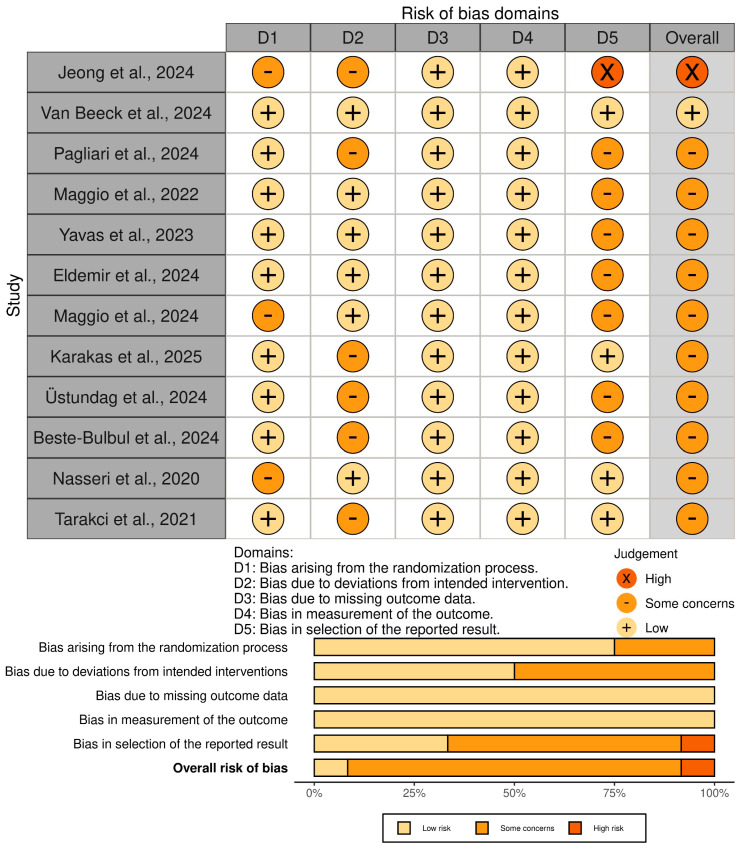
Risk of bias assessment with RoB2 tool [27,28,29,30,31,32,33,34,35,36,37,38].

Regarding the bias analysis using RoB2 [23,24,25], 1 study showed a high risk of bias [27], 1 a low risk [28], and 10 a moderate risk [29,30,31,32,33,34,35,36,37,38]. Regarding the sections with the highest risk of bias, sections D2 and D5 stand out. Section D2 refers to “Biases arising from deviations from the planned interventions,” which are common in physiotherapy research due to the difficulties of blinding interventions [40], even more so in the context of telerehabilitation. Section D5 refers to “Biases arising from the selection of the reported outcome,” with biases mainly found due to the lack of a published protocol reporting the pre-established statistical analysis prior to the trial. This analysis is presented in Figure 2.

According to the Oxford Classification of the level of evidence and grade of recommendation [26], eight trials obtained 1B-A results [28,29,30,31,32,33,36,38], as they were RCTs of good methodological quality with highly recommendable interventions and demonstrated evidence. The study by Jeong et al. [27] received a 2B-A rating, as it was an RCT of lower methodological quality, but it used an easily applicable intervention with benefits demonstrated by higher-quality studies. Two studies obtained a score of 2B-B [33,34], as they were trials with greater methodological limitations but a recommendable intervention. Nasseri et al. [37] obtained a score of 2B-C due to certain methodological limitations and no large effect sizes obtained with their interventions (Table 4).

## 5. Discussion

This review’s results indicate that TR has a positive impact on PwMS compared to no treatment, and it achieves similar or superior outcomes to conventional in-person treatment in terms of health-related QoL, thereby overcoming some common barriers to treatment.

The chronic course of MS, its progressive disability, and associated costs make it necessary to search for cost-effective strategies for the treatment of PwMS. The costs related to the treatment and management of MS in Spain range from EUR 10,486 to 27,217 per patient per year, depending on the severity [41]. Furthermore, direct non-healthcare costs, borne by patients, vary between EUR 454 and 25,850 annually, according to a study conducted by the Spanish Society of Neurology (SEN), Multiple Sclerosis Spain (EME), and the IESE Business School [41]. TR therefore appears to be a very interesting tool, improving the cost-effectiveness of treatment in populations with neurological problems and other conditions [42,43], enabling therapists to manage larger groups, and thereby making therapy accessible to a greater number of patients [36].

To our knowledge, previous systematic reviews have examined the effectiveness of different TR strategies in various aspects of PwMS [44,45,46]. Our results are in line with their findings, highlighting the need to create more specific protocols to study the clinical characteristics of each TR program and thus improve comparability. Despite this, as far as we know, only two reviews have used direct QoL measures as primary outcome measures [45,46]. Najafi et al. [46] found, in their systematic review with meta-analysis, a significant effect size in favor of tele-exercise in improving QoL of PwMS, using 13 RCTs for the meta-analysis. However, the authors already indicated the presence of great heterogeneity and a small effect size. On the other hand, Santos-Nascimento et al. [45] focused their review on the use of virtual reality, also showing positive effects in favor of the intervention with TR, although they used only two studies to carry out the meta-analysis on QoL [47,48], both showing significant results supporting the use of virtual reality in PwMS with low levels of impairment and with moderate previous levels of QoL. Our systematic review, although without a meta-analysis, offers a wider variety of TR interventions and ranges of patient involvement, providing a more up-to-date view of the treatment possibilities offered by these TR tools and chrono-scheduling and monitoring modes. It also provides a detailed analysis of the included articles, as well as the risk of bias, levels of evidence, and grades of recommendation.

Regarding the scales used to measure “participation” [8,9,11] in PwMS, QoL scales, the primary outcome measure of this review, stand out. MSQoL-54 was the most frequently used scale in a total of six trials [27,29,30,32,33,35]. Significantly greater improvements in MSQoL-54 were found in the TR group on four occasions [29,31,34,35], while similar results were observed between groups in the remaining eight trials [28,30,32,33,36,37,38], demonstrating the effectiveness of both TR and presential rehabilitation in improving QoL in PwMS and their superiority to no intervention [31,32]. A relevant aspect of social participation in PwMS [8,9,11] is the pelvic floor health-related QoL [11]. This was studied by Yavas et al. [31] using the “International Consultation of Incontinence Questionnaire-Short Form-Quality of Life” (ICIQ-UI-SF-QoL), and they obtained similar results between the TR and face-to-face groups, with significantly greater improvements in the TR group compared to the group that received no treatment [31]. Symptoms of sexual dysfunction are present in 40–80% of women and 50–90% of men with MS, negatively affecting their QoL [49] and significantly impacting their mental health and psychological well-being [50]. In this sense, previous systematic reviews have demonstrated the effectiveness of TR on pelvic floor pathologies [51] but, as far as we know, its impact on QoL of PwMS has not been directly studied, despite its high prevalence [49,52,53]. Despite this, trials included in this review show promising results in this area [31,33,36], and previous cohort studies show evidence on how depression and illness perception may affect sexual dysfunction in PwMS [54]. Therefore, TR may be as effective as conventional in-person rehabilitation for improving participation-related aspects of PwMS, and more effective than no treatment.

The most commonly used scales for assessing “body structures and functions” and “activity” [8,9,11], were the “Fatigue Severity Scale” (FSS) [29,32,38] and the “Nine Hole Peg Test” (NHPT) [28,29,37], respectively. Both fatigue and manual dexterity are related to the patient’s ability to perform activities of daily living, and therefore affect their QoL [54,55]. The studies included in this review demonstrated a range of clinical effects, consistent with the other outcome measures analyzed. In the NHPT, van Beeck [28] and Pagliari [29] found significant improvements in the TR group compared to upper limb strength training [28] and conventional treatment [29], and Nasseri [37] found improvements in both groups, without showing significant differences. Regarding the FSS, Pagliari [29] showed significant improvements in the TR group, Tarakci [38] found significant differences in favor of the in-person control group, and Eldemir [32] obtained improvements in the TR group, while the control worsened since they did not undergo treatment. This could indicate that therapist guidance may be necessary in the treatment of PwMS. Notably, Pagliari et al. also reported significant improvements in the TR groups for both scales [29], probably due to the fact that they carried out the most training sessions, with 30 in total. In contrast, the greater improvements in the FSS in the face-to-face group in the trial carried out by Tarakci et al. [38] is explained by the authors both by the difficulty of remotely measuring fatigue and by the possible lack of motivation due to not feeling part of a rehabilitation program. This is consistent with the results of other trials, such as the one conducted by Maggio et al. in 2023 [56], which showed greater improvements in the psychological component of QoL of PwMS with the introduction of guided real-time supervisions, with social support also being a predictor of health-related QoL in PwMS [57]. Therefore, TR appears to be just as effective as in-person rehabilitation in improving aspects related to body structures, functions, and activity in people with multiple sclerosis, and it is also more effective than no treatment.

It is worth noting that the study by Nasseri et al. [37], the two by Maggio et al. [30,33], and the one by Jeong et al. [27] are the only ones that present patients with EDSS levels > 6, with the trial conducted by Nasseri [37] being the only one whose patients only showed progressive forms of MS; therefore, the effects of TR may have been influenced by the greater degree of disability or by the distinct disease course. In these trials, a trend towards better results in the TR group was observed as the EDSS of the included patients increased, with significant differences in favor of TR found in the trials performed by Jeong et al. [27] and Maggio et al. [30,33]. Despite this, only Jeong et al. [27] used a sample of patients with EDSS > 5.5, while Maggio et al. and Nasseri et al. also included patients with lower levels of impairment [30,33,37], so the introduction of patients with different levels of disability makes it difficult to draw conclusions. A trial conducted by Faramarzi et al. in 2020 [58] included exercise in PwMS, dividing them into groups according to their disability levels (EDSS < 4.5 vs. 4.5–6 vs. EDSS > 6) and found no significant differences in functional or strength improvements. This, unlike what our results suggest, could be due to the fact that rehabilitation was carried out in person and the lack of use of QoL scales, in addition to the use of the 6-Minute Walking Test and the Timed up and Go Test as functional outcomes. These scales may not be the most appropriate for measuring functional capacity in patients with walking problems, as would be the case in patients with EDSS > 6. Furthermore, the high disability group had the lowest number of participants, which could also affect the statistical power of the effects obtained. On the other hand, observing the samples of Üstundag et al. [35] and Yavas et al. [31], the only two studies that obtained significantly greater improvements in the TR group and provided data on the types of MS found among their patients, we found mostly patients with RRMS (100% in the study by Üstundag et al. [35] and 87% in that of Yavas et al. [31]), but with low to moderate degrees of functional limitation, so both the degree of disability and the type of MS evolution could be modifying factors of the effectiveness of TR. Despite this, a trial conducted by Najafi et al. in 2023 [59] studied the effects of two yoga- and Pilates-based TR programs in people with RRMS versus the same programs in people with SPMS with EDSS levels < 6, without finding significant differences in improvements in QoL between groups. Combining these results with ours, one could hypothesize that the degree of disability could be a more relevant factor in terms of the effects obtained through TR programs than the MS progression type.

Regarding the clinical advantages provided by TR, it is relevant to mention treatment adherence [60], the possibility of generating greater motivation [61,62] and the ability to reduce the barriers that these patients may encounter [63]. TR, particularly under real-time supervision, can be an effective tool for improving program adherence, as it enables and facilitates patient monitoring [31,38], leading to higher adherence rates than conventional rehabilitation, as demonstrated in studies such as that by Pagliari et al. [29]. Furthermore, systematic reviews have shown that gamification, which can be easily integrated with TR systems, increases motivation and adherence to treatment in children and adolescents with neurological conditions [64], and has also shown promising results in the treatment of fatigue in PwMS [65,66]. Patients often face treatment barriers, including limited access to specialized care, poor communication from medical professionals, inadequate comprehensive care, and challenges posed by the MS symptoms themselves [67]. Therefore, TR could be a useful intervention to reduce patient-perceived barriers to treatment, primarily those related to geographical factors or significant travel difficulties due to the disease.

An additional perspective to take into account regarding TR is the potential role of cognitive re-education components embedded within telerehabilitation programs. Recent evidence suggests that digital interventions, such as computer-based cognitive training, semi-immersive or virtual reality tasks, and metacognitive or self-generation strategies, can mediate improvements in QoL by enhancing processing speed, attention, executive control, and self-efficacy, while also mitigating depressive and anxiety symptoms. These mechanisms may foster better functional participation and perceived well-being, providing a plausible pathway linking symptom management and activity gains to broader QoL outcomes, consistent with the ICF framework [68]. TR may, in fact, improve QoL by embedding these cognitive re-education elements that reinforce information processing, attentional control, executive functioning, and self-regulatory skills. The recent work on cognitive re-education in multiple sclerosis supports this view [68], showing consistent cogni-tive gains alongside variable but notable improvements in mental-health–related QoL when programs are suffi-ciently engaging, supervised, and tailored. VR- and computer-based training modalities have reported concurrent benefits in memory and attention, as well as in depressive symptoms, functional status, and mental QoL. Such pathways align with the ICF model employed in this review: gains at the level of body functions (cognition, mood) and activities (efficiency, self-management) may translate into enhanced participation and overall QoL, particu-larly when interventions incorporate metacognitive strategies, self-generation learning, gamification and feedback, and real-time professional oversight that sustain motivation and adherence. Nonetheless, effects can be heteroge-neous and sometimes transient, underscoring the importance of individualized goal-setting, adequate intensity and supervision, and follow-up strategies, such as booster or hybrid programs, to maintain benefits over time.

Finally, the analyzed trials demonstrated moderate to high quality on the CASP scale, with moderate to low risk of bias, primarily limited by domains D2 and D5 of the RoB2. A total of 8 of the 12 included trials had a level of evidence of 1B and a grade of recommendation of A, reinforcing the robustness and clinical applicability of the conclusions of this systematic review.

Our review is not free of limitations. Firstly, the search was limited to the last 10 years and the English and Spanish languages, possibly limiting the amount of information obtained. Our results cannot be directly extrapolated to other populations with different neurological pathologies and are restricted to patients with EDSS scores below 7.5, so these effects may not be replicated in subjects with greater impairment. The varying methods used in TR programs, the diverse characteristics of the study samples, and the different protocol durations make it challenging to draw homogeneous conclusions and conduct a meta-analysis. Lastly, only two trials included post-intervention follow-up, after 4 weeks [34] and 12 weeks [28]; therefore, it cannot be stated that the short-term results obtained translate into medium- or long-term improvements in the QoL of PwMS.

## 6. Conclusions

TR platforms and programs improve health-related QoL in PwMS compared to no intervention, with similar effects to in-person rehabilitation in patients with EDSS levels < 6. Furthermore, TR appears to be superior to in-person rehabilitation in people with EDSS levels between 5.5 and 7.5. These platforms could also serve as a tool to promote treatment adherence in home-based programs and eliminate some of the barriers encountered by MS patients.

Future work using TR platforms in PwMS with varying degrees of disability and including follow-up evaluations is needed to assess the potential medium- and long-term benefits of these systems on QoL in PwMS.

## Figures and Tables

**Table 1:** Search strategies.

Database	Search Strategy
**PubMed**	(“Telerehabilitation” [MeSH Terms] OR “telerehabilitation” [Title/Abstract] OR “virtual rehabilitation” [Title/Abstract] OR “rehabilitation virtual” [Title/Abstract] OR “tele rehabilitation” [Title/Abstract] OR “Tele rehabilitation” [Title/Abstract] OR “remote rehabilitation” [Title/Abstract] OR “rehabilitation remote” [Title/Abstract] OR “Neurorehabilitation” [Title/Abstract] OR “Neurophysiotherapy” [Title/Abstract] OR “physiotherapy” [Title/Abstract] OR “physical therapy” [Title/Abstract] OR “technolog health care” [Title/Abstract] OR “Health Technology” [Title/Abstract] OR “Remote Sensing Technology” [MeSH Terms] OR “remote sens” [Title/Abstract] OR “Virtual Reality” [MeSH Terms] OR “reality virtual” [Title/Abstract] OR “Mobile Applications” [MeSH Terms] OR “application mobile” [Title/Abstract] OR “Mobile Apps” [Title/Abstract] OR “Smartphone Apps” [Title/Abstract] OR “app” [Title/Abstract] OR “apps” [Title/Abstract] OR “wii fit” [Title/Abstract] OR “x-box” [Title/Abstract] OR “nintendo switch” [Title/Abstract]) AND (“Multiple Sclerosis” [MeSH Terms] OR “Multiple Sclerosis” [Title/Abstract] OR “Sclerosis Multiple” [Title/Abstract] OR “ms multiple sclerosis” [Title/Abstract] OR “Sclerosis Disseminated” [Title/Abstract] OR “Disseminated Sclerosis” [Title/Abstract]) AND (“Quality of Life” [MeSH Terms] OR “Life Quality” [Title/Abstract] OR “health related quality of life” [Title/Abstract] OR “health related quality of life” [Title/Abstract] OR “wellbeing” [Title/Abstract] OR “happiness” [Title/Abstract] OR “welfare” [Title/Abstract] OR “well-being” [Title/Abstract] OR “comfort” [Title/Abstract]) Filters: in the last 10 years, Clinical Trial, Clinical Trial, Phase I, Clinical Trial, Phase II, Clinical Trial, Phase III, Clinical Trial, Phase IV, Controlled Clinical Trial, Randomized Controlled Trial
**EMBASE**	(‘telerehabilitation’/exp OR ‘telerehabilitation’:ti,ab,kw OR ‘virtual rehabilitation’:ti,ab,kw OR ‘rehabilitation virtual’:ti,ab,kw OR ‘tele rehabilitation’:ti,ab,kw OR ‘tele rehabilitation’:ti,ab,kw OR ‘remote rehabilitation’:ti,ab,kw OR ‘rehabilitation remote’:ti,ab,kw OR ‘neurorehabilitation’:ti,ab,kw OR ‘neurophysiotherapy’:ti,ab,kw OR ‘physiotherapy’:ti,ab,kw OR ‘physical therapy’:ti,ab,kw OR ‘technolog health care’:ti,ab,kw OR ‘health technology’:ti,ab,kw OR ‘remote sensing’/exp OR ‘remote sens’:ti,ab,kw OR ‘virtual reality’/exp OR ‘reality virtual’:ti,ab,kw OR ‘mobile application’/exp OR ‘application mobile’:ti,ab,kw OR ‘mobile apps’:ti,ab,kw OR ‘smartphone apps’:ti,ab,kw OR ‘app’:ti,ab,kw OR ‘apps’:ti,ab,kw OR ‘wii fit’:ti,ab,kw OR ‘x-box’:ti,ab,kw OR ‘nintendo switch’:ti,ab,kw) AND (‘multiple sclerosis’/exp OR ‘multiple sclerosis’:ti,ab,kw OR ‘sclerosis multiple’:ti,ab,kw OR ‘ms multiple sclerosis’:ti,ab,kw OR ‘sclerosis disseminated’:ti,ab,kw OR ‘disseminated sclerosis’:ti,ab,kw) AND (‘quality of life’/exp OR ‘life quality’:ti,ab,kw OR ‘health related quality of life’:ti,ab,kw OR ‘wellbeing’:ti,ab,kw OR ‘happiness’:ti,ab,kw OR ‘welfare’:ti,ab,kw OR ‘well-being’:ti,ab,kw OR ‘comfort’:ti,ab,kw) AND (2015:py OR 2016:py OR 2017:py OR 2018:py OR 2019:py OR 2020:py OR 2021:py OR 2022:py OR 2023:py OR 2024:py OR 2025:py) AND [embase]/lim NOT ([embase]/lim AND [medline]/lim) AND (‘clinical protocol’/de OR ‘clinical trial’/de OR ‘clinical trial protocol’/de OR ‘clinical trial topic’/de OR ‘controlled clinical trial’/de OR ‘multicenter study’/de OR ‘randomized controlled trial’/de OR ‘randomized controlled trial topic’/de)
**Dialnet**	(telefisioterapia OR telerrehabilitación OR telemedicina OR teleasistencia) AND “esclerosis múltiple” AND “calidad de vida”
**PeDro**	Abstract & Title: (Telerehabilitation OR Telemedicine OR Teleassistance OR “Virtual Medicine” OR “Remote Care”) AND “multiple sclerosis” AND “quality of life”
**Web of Science**	((“Multiple sclerosis” [Mesh]) OR (“Sclerosis multiple”) OR (“Disseminated sclerosis”) (Topic)) AND ((“Telerehabilitation” [Mesh]) OR (“Virtual rehabilitation”) OR (“Remote rehab”) OR (“Remote physical therap”) (Topic)) AND ((“Quality of life” [Mesh]) OR (“Life quality”) OR (“Health-related Quality of Life”) (Topic)) AND 1 January 2015/27 September 2025 (Publication Date)

**Table 2:** Analysis of the main characteristics of the included studies.

**Author**	Sample	Characteristics	Age and Time Since Diagnosis	Interventions	Outcomes	Results
Jeong et al. (2024) [27]	45 participants IG: 29 (23 F/6 M) CG: 16 (10 F/6 M)	EDSS: ≥5.5 y ≤7.5 inter-group differences not reported IG: 6 Afro-Americans, 9 Caucasians, 1 other. Self-reported severity: mild (2), moderate (8), severe (6) CG: 8 Afro-Americans, 2 Asiatic, 18 Caucasians, 1 other. Self-reported severity: none (1), mild (6), moderate (17), severe (5) TYPES: Not reported	IG: 57.8 ± 11.9 Disease duration: 2.5–49.6 years CG: 56.0 ± 12.8 Disease duration: 1–50 years	Treatment not reported TR METHOD “Home-TR system” SUPERVISION PT	MSQoL-54.	Improvements are shown in all subscales in both groups IG showed significantly greater improvements in pain (F = 4.301; *p* = 0.044) and cognitive function (F = 5.053; *p* = 0.03)
van Beeck et al. (2024) [28]	48 participants IG: 26 (21 F/5 M) CG: 22 (15 F/7 M)	EDSS: IG: 2.98 ± 1.81 CG: 3.03± 2.23 TYPES: IG: RRMS 65%, PPMS 23%, SPMS 12% CG: RRMS 68%, PPMS 23%, SPMS 9%	IG: 50.84 ± 14.84 Disease duration: 12.33 ± 8.23 years CG: 48.40 ± 14.61 Disease duration: 11.69 ± 9.57 years	IG: Tablet-based exercises CG: Upper limb and hand strength training 4 weeks, 5 sessions per week, 30’ per session. 12th week follow-up TR METHOD: Tablet app. SUPERVISION: Not reported.	AMSQ NHPT JAMAR CRT MSIS-29	IG showed significantly greater improvements in physical (−6.29; *p* = 0.001; 95%CI-9.88; −2.27) and psychological domains (–6.93; *p* = 0.023; 95%CI-12.82; –1.04) of MSIS-29 No statistical differences were found in MSIS-29 improvements between groups
Pagliari et al. (2024) [29]	70 participants GE: 35 GC: 35 Sex not reported	EDSS: ≤6.5 TYPES: Not reported	Age between 25 and 70 years. Inter-group differences not reported. Time since diagnosis not reported.	GE: Cognitive and motor treatment with VR GC: Conventional PT with motor and cognitive treatment; 6 weeks, 5 sessions per week, 45’ per session. TR METHOD VR system SUPERVISION “Offline monitoring”. System collects data that therapists review.	MSQoL-54 MSWS-12 BDI FSS RESE STAI BBT Mini-BESTest 9-HPT MoCA SDMT SRT-LTS SRT-CLTR 10/36 SPART PASAT 3s SRT-DR D-10/36-SPART WLG	IG showed significantly more adherence (*p* = 0.045) than CG. IG showed significantly greater improvements in physical domain of MSQoL-54 than CG (*p* = 0.039).
Maggio et al. (2022) [30]	60 participants IG: 30 (12 F/18 M) CG: 30 (17 F/13 M)	EDDS: ≤7 TYPES: RRMS and SPMS (n° not reported)	IG: 51.9 ± 9.9 CG: 48.2 ± 12.2 Disease duration not reported	IG: Exercises with BTS-Nirvana system CG: traditional exercise TR METHOD: BTS-Nirvana SUPERVISION: Physical therapist	MoCA PASAT-2 SPART ROCF BDI TUG Tinetti Control Trunk Test MSQoL-54	Both groups showed significant improvements MSQOL-54, without statistical significance. Only IG showed significant improvements in visual perception and visuospatial skills, visual short-term memory, working memory, executive functions, information processing speed, sustained attention and TUG.
Yavas et al. (2023) [31]	45 participants IG1: 15 (12 F/3 M) IG2: 15 (12 F/3 M) CG: 15 (12 F/3 M)	EDSS (average): 3.67–3.93 TYPES: RRMS 80–87%	IG1: 40.1 Disease duration: 13.97 years IG2: 48.6 Disease duration: 13.49 years CG: 45.3 Disease duration: 18 years	IG1: TR exercises with supervision twice per week IG2: Same TR program without supervision CG: Usual care; 12 weeks, follow-ups every 4 weeks TR METHOD: Skype videocall SUPERVISION: PT	Use of compresses Number of voids ICIQ-UI-SF OAB-v8 MSISQ-19 EuroQoL ICIQ-UI-SF-QoL. HADS BICAMS 3-day voiding diary Reported adherence and satisfaction	IG1 showed greater adherence, satisfaction, perceived continence and QoL Both IGs showed improvements in number of leaks and compresses compared to CG, without differences between them. All groups improved sexuality, anxiety, and depression without statistical significance.
Eldemir et al. (2024) [32]	30 participants IG: 15 (14 F/1 M) CG: 15 (14 F/1 M)	EDSS: 0–5 Inter-group differences not reported TYPES: Not reported	IG: 41 ± 7.82 CG: 38.4 ± 10.86 Disease duration not reported	IG: Remote Pilates CG: No treatment; 6 weeks, 3 sessions per week, 1 h per session TR METHOD: Videoconference SUPERVISION: PT	MSQoL-54 FSS FIS Grip strength Side-bridge test Biering–Sorensen test Trunk flexor test Prone bridge test BBS 6 MWT G-walk Sensor system	IG showed significantly greater improvements in strength outcomes, functional balance, gait speed, functional capacity, and both physical and mental MSQoL-54 subscales (*p* < 0.001), without changes in other spatiotemporal parameters. CG showed a significant deterioration in FSS, without changes in other fitness and QoL outcomes (physical MSQoL-54 *p* = 0.198; mental MSQoL-54 *p* = 0.329).
Maggio et al. (2024) [33]	70 participants IG: 35 (21 F/14 M) CG: 35 (19 F/16 M)	EDSS: <7 TYPES: RRMS and SPMS Inter-group differences not reported	IG: 46.09 ± 10.4 CG: 52.46 ± 8.99 Disease duration not reported	IG: SI-VR with “BTS-Nirvana” system CG: Traditional presential rehabilitation TR METHOD: VR with BTS-Nirvana System SUPERVISION: PT	MSQoL-54.	IG showed significant improvements in all domains of MSQoL-54 (*p* < 0.001), except for pain and role limitations. CG showed no significant changes in QoL.
Karakas et al. (2025) [34]	32 participants IG: 16 (12 F/4 M) CG: 16 (12 F/4 M)	EDSS: <3 TYPES: Not reported	IG: 37 (32.5–40) Disease duration: 10.3 ± 7.1 years CG: 37.5 (30.2–43.7) Disease duration: 7.75 ± 4.8 years	IG: Motor imagery CG: Continued their usual treatment 8 weeks TR METHOD: “Neuro Orthopedic Institute Recognize app” and Google Meet videoconference SUPERVISION: PT	VAS (pain) PDQ KVIQ Mental chronometry MFIS HADS MusiQol PSQI ESS SDMT CVLT-II BVMT-R	IG showed significantly greater improvements in pain (*p* < 0.05) and depression (*p* < 0.05) compared to CG. No significant inter-group differences were found in KVIQ scores or mental chronometry (*p* > 0.5).
Üstündag et al. (2024) [35]	63 participants IG: 31 (21 F/10 M) CG: 32 (24 F/8 M)	EDSS: ≤6 Inter-group differences not reported TYPES: IG: 28 RRMS, 3 SPMS CG: 28 RRMS, 4 SPMS	IG: 33.90 ± 8.36. Disease duration: 8.26 ± 5.67 years CG: 39.16 ± 8.78. Disease duration: 10.38 ± 5.62 years	IG: App CG: Standard care; 12 weeks TR METHOD Mobile app with direct messages SUPERVISION PT, neurologist, and nurse	MS-RS. MSQoL-54. MS-TAQ.	MS-RS and MS-TAQ decreased in IG and increased in CG, but significant differences were not found. IG showed significantly greater improvements in MSQoL-54 (*p* < 0.05).
Beste Bulbul et al. (2024) [36]	42 participants IG: 21 CG: 21 All participants were F	EDSS: <6.5 Inter-group differences not reported TYPES: Not reported	18–65 years Inter-group differences and disease durations not reported	IG: Pelvic floor TR training and lifestyle advice CG: Lifestyle advice; 8 weeks, 1 supervised session per week (unsupervised other 4 sessions), 20’ per session	KHQ OAB-V8 ICIQ-SF	IG showed significantly greater improvements in OAB-V8 score (*p* < 0.001), ICIQ-SF (*p* = 0.013), nocturia (*p* = 0.02), general health perception (*p* = 0.004), incontinence impact (*p* = 0.006), role limitations (*p* < 0.001), social limitations (*p* < 0.001), emotional problems (*p* < 0.001), subjective perception of improvement (*p* < 0.001) and satisfaction (*p* = 0.001).
				TR METHOD Weekly videocall plus rutinary check-up via email SUPERVISION PT		
Nasseri et al. (2020) [37]	38 participants IG: 18 (9 F/9 M) CG: 20 (11 F/9 M)	EDSS: IG: 3.5 (2.5–6.0) CG: 3.5 (3.0–6.0) TYPES: PPMS or SPMS	IG: 49.6 ± 8.5. Disease duration: 13.1 ± 5.6 years CG: 52.5 ± 7.3. Disease duration: 20.1 ± 13.0 years	IG: App CG: Printed sheet with exercise recommendations 12 weeks TR METHOD EBPI smartphone app SUPERVISION Neurologist	HAQUAMS MSWS-12 Godin Leisure Time Exercise Questionnaire App accelerometry (steps/min, diary METs and % of moderate physical activity) 2MWT 6MWT T25 FW Timed Tandem Walk. NHPT 5 TSST Symbol Digit Modalities Test	No significant differences were found between groups in MSWS (*p* = 0.82), HAQUAMS (*p* = 0.71), or NHPT (*p* = 0.4).
Tarakci et al. (2021) [38]	30 participants IG: 15 (11 F/4 M) CG: 15 (12 F/3 M)	EDSS: IG: 3.40 ± 1.53 CG: 3.46 ± 1.31 TYPES: Not reported	CG: 41 ± 11.09. Disease duration: 6.20 ± 3.96 years IG: 39.46 ± 10.59. Disease duration: 8.86 ± 4.50 years	IG: TR supervised exercise program with videocalls CG: Presential exercise program; 12 weeks TR METHOD: Videocall SUPERVISION: PT	FIM NHP-I FSS QoLS	Both groups showed significant improvements in all outcomes measured. CG showed significantly greater improvements in FSS (*p* = 0.001) and NHPT (*p* = 0.44) compared to IG. No significant differences were found between groups in FIM (*p* = 0.098) and QoL (*p* = 0.256).

10/36 SPART: 10/36 Special Recall Test, 2MWT: Two-Minute Walk Test, 5TSST: Five-Time Sit to Stand Test, 6MWT: Six-Minute Walk Test, AMSQ: Arm Function in Multiple Sclerosis Questionnaire, BBS: Berg Balance Scale, BBT: Box and Blocks Test, BDI: Beck Depression Inventory, BICAMS: Brief International Cognitive Assessment for Multiple Sclerosis, BVMT-R: Brief Visuospatial Memory Test-Revised, CG: control group CRT: Coin Rotation Test, CSQ-8: Client Satisfaction Questionnaire, CVLT-II: California Verbal Learning Test—Second Edition, D-10/36-SPART: D-10/36 Special Recall Test—Delayed Recall, EBPI: Evidence-Based Patient Information EDSS: Expanded Disability Status Scale, ESS: Epworth Sleepiness Scale, F: Female, FIM: Functional Independence Measure, FIS: Fatigue Impact Scale, FSFI: Female Sexual Function Index, FSS: Fatigue Severity Scale, HADS: Hospital Anxiety and Depression Scale, HAQUAMS: Hamburg Quality of Life Questionnaire, HRQoL: Health-Related Quality of Life, ICIQ-UI-SF: International Consultation on Incontinence Questionnaire—Short Form, ICIQ-UI-SF-QoL: International Consultation on Incontinence Questionnaire—Short Form—Quality of Life, IG: Intervention Group, KHQ: King’s Health Questionnaire, KVIQ: Kinesthetic and Visual Imagery Questionnaire, M: male, MoCA: Montreal Cognitive Assessment, MFIS: Modified Fatigue Impact Scale, MSIS-29: Multiple Sclerosis Impact Scale-29, MSISQ-19: Multiple Sclerosis Intimacy and Sexuality Questionnaire, MSQoL-54: Multiple Sclerosis Quality of Life-54, UE: upper extremity, MS-RS: Multiple Sclerosis Rating Scale, MS-TAQ: Multiple Sclerosis Treatment Adherence Questionnaire, MSWS-12: 12-item Multiple Sclerosis Walking Scale, MusiQoL: Multiple Sclerosis International Quality of Life, NASA-TLX: NASA Task Load Index, NHP: Nottingham Health Profile, NHPT: Nine Hole Peg Test, NHP-I: Nottingham Health Profile—Part I, OAB-V8: Overactive Bladder Awareness Tool, PASAT 3s: Paced Auditory Serial Addition Test at 3 s, PDQ: Pain Detect Questionnaire, PSQI: Pittsburgh Sleep Quality Index, PT: physical therapy, QoLS: Quality of Life Scale, RESE: Regulatory Emotional Self-Efficacy Scale, ROCF: Rey–Osterrieth Complex Figure, SDMT: Symbol Digit Modalities Test, SI-VR: semi-immersive visual reality, SPART: Spatial Recall Test, SRT-CLTR: Selective Reminding Test—Consistent Long-Term Recall, SRT-DR: Selective Reminding Test—Delayed Recall, SRT-LTS: Selective Reminding Test—Long-Term Storage, STAI: State–Trait Anxiety Inventory, T25FW: Timed 25-Foot Walk, TMT: Trail Making Test, TR: Telerehabilitation, VAS: Visual Analogic Scale, WEIMuS: Würzburg Fatigue Inventory for MS, WLG: Word List Generation. Underlined and bold scales show results on quality of life.

**Table 3:** Methodological quality of the included articles.

Author	1	2	3	4	5	6	7	8	9	10	11	Total
Jeong et al. (2024) [27]	1	1	0	0	1	0	0	0	1	1	1	6/11
van Beeck et al. (2024) [28]	1	1	0	1	1	1	1	0	1	1	1	9/11
Pagliari et al. (2024) [29]	1	1	0	1	1	0	1	0	1	1	1	8/11
Maggio et al. (2022) [30]	1	1	0	1	1	0	1	0	1	1	0	7/11
Yavas et al. (2023) [31]	1	1	0	1	1	0	1	0	1	1	1	8/11
Eldemir et al. (2024) [32]	1	1	0	1	1	0	1	0	1	1	0	7/11
Maggio et al. (2024) [33]	1	1	0	1	1	0	1	0	1	1	0	7/11
Karakas et al. (2025) [34]	1	1	0	1	1	1	1	1	1	1	1	10/11
Üstündag et al. (2024) [35]	1	1	0	1	1	0	1	0	1	1	0	7/11
Beste Bulbul et al. (2024) [36]	1	1	0	1	0	0	1	1	1	1	1	8/11
Nasseri et al. (2020) [37]	1	1	0	1	1	1	1	1	1	1	1	10/11
Tarakci et al. (2021) [38]	1	1	0	1	1	1	1	0	1	1	1	9/11

**Table 4:** Levels of evidence and grades of recommendation.

Study	Level of Evidence	Grade of Recommendation
Jeong et al. (2024) [27]	2B	A
Van Beek et al. (2024) [28]	1B	A
Pagliari et al. (2024) [29]	1B	A
Maggio et al. (2022) [30]	1B	A
Yavas et al. (2023) [31]	1B	A
Eldemir et al. (2024) [32]	1B	A
Maggio et al. (2024) [33]	2B	B
Karakas et al. (2025) [34]	2B	B
Üstündag et al. (2024) [35]	1B	A
Beste Bulbul et al. (2024) [36]	1B	A
Nasseri et al. (2020) [37]	2B	C
Tarakci et al. (2021) [38]	1B	A

## Data Availability

No new data were created or analyzed in this study. Data sharing is not applicable to this article.

## Supplementary Materials

The following supporting information can be downloaded at https://www.mdpi.com/article/10.3390/neurosci6040103/s1: Supplementary Material S1: PRISMA checklist; Supplementary Material S2: ICF domain classification of scales; Supplementary Material S3: QoL scale descriptions.
