# Supplementary material for: Effects of Telerehabilitation Platforms on Quality of Life in People with Multiple Sclerosis: A Systematic Review of Randomized Clinical Trials
Source: NeuroSci. 2025 Oct 13;6(4):103. doi: 10.3390/neurosci6040103 (PMC12550922; doi:10.3390/neurosci6040103)
Supplement: Supplementary file 1 [file neurosci-06-00103-s001.zip › neurosci-3884752-supplementary.pdf]

PRISMA 2020 Checklist

Section and Topic	Item #	Checklist item	Location where item is reported
**TITLE**			
Title	1	Identify the report as a systematic review.	Yes; p1. Title
**ABSTRACT**			
Abstract	2	See the PRISMA 2020 for Abstracts checklist.	Yes; p.2 Abstract
**INTRODUCTION**			
Rationale	3	Describe the rationale for the review in the context of existing knowledge.	Yes; p.4 Introduction
Objectives	4	Provide an explicit statement of the objective(s) or question(s) the review addresses.	Yes; p.5 Purpose
**METHODS**			
Eligibility criteria	5	Specify the inclusion and exclusion criteria for the review and how studies were grouped for the syntheses.	Yes; p.6 Methods-Study selection
Information sources	6	Specify all databases, registers, websites, organisations, reference lists and other sources searched or consulted to identify studies. Specify the date when each source was last searched or consulted.	Yes; p.6 Methods-Search databases
Search strategy	7	Present the full search strategies for all databases, registers and websites, including any filters and limits used.	Yes; p.15 Table 1
Selection process	8	Specify the methods used to decide whether a study met the inclusion criteria of the review, including how many reviewers screened each record and each report retrieved, whether they worked independently, and if applicable, details of automation tools used in the process.	Yes; p.7 Methods-Result analysis
Data collection process	9	Specify the methods used to collect data from reports, including how many reviewers collected data from each report, whether they worked independently, any processes for obtaining or confirming data from study investigators, and if applicable, details of automation tools used in the process.	Yes; p.7 Methods-Result analysis
Data items	10a	List and define all outcomes for which data were sought. Specify whether all results that were compatible with each outcome domain in each study were sought (e.g. for all measures, time points, analyses), and if not, the methods used to decide which results to collect.	Yes; p.5 Methods-Outcomes
	10b	List and define all other variables for which data were sought (e.g. participant and intervention characteristics, funding sources). Describe any assumptions made about any missing or unclear information.	Partially; p.17 Table 2
Study risk of bias assessment	11	Specify the methods used to assess risk of bias in the included studies, including details of the tool(s) used, how many reviewers assessed each study and whether they worked independently, and if applicable, details of automation tools used in the process.	Yes; p.8 Methods- Risk of Bias
Effect measures	12	Specify for each outcome the effect measure(s) (e.g. risk ratio, mean difference) used in the synthesis or presentation of results.	Partial; p.7 Methods-Outcomes (only narrative synthesis)
Synthesis	13a	Describe the processes used to decide which studies were eligible for each synthesis (e.g. tabulating the study intervention characteristics	Yes; p.6

PRISMA 2020 Checklist

Section and Topic	Item #	Checklist item	Location where item is reported
methods		and comparing against the planned groups for each synthesis (item #5)).	Methods-Study selection
	13b	Describe any methods required to prepare the data for presentation or synthesis, such as handling of missing summary statistics, or data conversions.	No
	13c	Describe any methods used to tabulate or visually display results of individual studies and syntheses.	Partially; p.17 Table 2 and narrative synthesis
	13d	Describe any methods used to synthesize results and provide a rationale for the choice(s). If meta-analysis was performed, describe the model(s), method(s) to identify the presence and extent of statistical heterogeneity, and software package(s) used.	Partially (narrative synthesis)
	13e	Describe any methods used to explore possible causes of heterogeneity among study results (e.g. subgroup analysis, meta-regression).	No
	13f	Describe any sensitivity analyses conducted to assess robustness of the synthesized results.	Yes; p8 Results-Methodological quality and Risk of bias
Reporting bias assessment	14	Describe any methods used to assess risk of bias due to missing results in a synthesis (arising from reporting biases).	No
Certainty assessment	15	Describe any methods used to assess certainty (or confidence) in the body of evidence for an outcome.	Yes; p.9 Methods-Oxford classification
**RESULTS**			
Study selection	16a	Describe the results of the search and selection process, from the number of records identified in the search to the number of studies included in the review, ideally using a flow diagram.	Yes; p.25 Figure 1
	16b	Cite studies that might appear to meet the inclusion criteria, but which were excluded, and explain why they were excluded.	No
Study characteristics	17	Cite each included study and present its characteristics.	Yes; p.17 Table 2
Risk of bias in studies	18	Present assessments of risk of bias for each included study.	Yes; p.26 Figure 2
Results of individual studies	19	For all outcomes, present, for each study: (a) summary statistics for each group (where appropriate) and (b) an effect estimate and its precision (e.g. confidence/credible interval), ideally using structured tables or plots.	Partially; Results + Table 2 (narrative synthesis)
Results of syntheses	20a	For each synthesis, briefly summarise the characteristics and risk of bias among contributing studies.	Yes; p6-8 Results, Table 2 and Figure 2
	20b	Present results of all statistical syntheses conducted. If meta-analysis was done, present for each the summary estimate and its precision	Yes; p. 17

Section and Topic	Item #	Checklist item	Location where item is reported
		(e.g. confidence/credible interval) and measures of statistical heterogeneity. If comparing groups, describe the direction of the effect.	Table 2
	20c	Present results of all investigations of possible causes of heterogeneity among study results.	Yes; p.8-11 Discussion
	20d	Present results of all sensitivity analyses conducted to assess the robustness of the synthesized results.	Yes; p.8 Results-Methodological quality and Risk of bias
Reporting biases	21	Present assessments of risk of bias due to missing results (arising from reporting biases) for each synthesis assessed.	No
Certainty of evidence	22	Present assessments of certainty (or confidence) in the body of evidence for each outcome assessed.	Partial; p.15 Results-Oxford classification
**DISCUSSION**			
Discussion	23a	Provide a general interpretation of the results in the context of other evidence.	Yes; p.8-12 Discussion
	23b	Discuss any limitations of the evidence included in the review.	Yes; p.12-Discussion (last paragraph)
	23c	Discuss any limitations of the review processes used.	Yes; p.12-Discussion (last paragraph)
	23d	Discuss implications of the results for practice, policy, and future research.	Yes; p.12-13 Conclussions
**OTHER INFORMATION**			
Registration and protocol	24a	Provide registration information for the review, including register name and registration number, or state that the review was not registered.	Yes; p.2 Abstract-Materials and Methods
	24b	Indicate where the review protocol can be accessed, or state that a protocol was not prepared.	Yes; p. 2 Abstract-Materials and Methods
	24c	Describe and explain any amendments to information provided at registration or in the protocol.	Yes (modifications not done)
Support	25	Describe sources of financial or non-financial support for the review, and the role of the funders or sponsors in the review.	Yes; p.2 Funding
Competing	26	Declare any competing interests of review authors.	Yes; p.2

Section and Topic	Item #	Checklist item	Location where item is reported
interests			Conflict of interest
Availability of data, code and other materials	27	Report which of the following are publicly available and where they can be found: template data collection forms; data extracted from included studies; data used for all analyses; analytic code; any other materials used in the review.	Yes; Indications of supplementary materials

From: Page MJ, McKenzie JE, Bossuyt PM, Boutron I, Hoffmann TC, Mulrow CD, et al. The PRISMA 2020 statement: an updated guideline for reporting systematic reviews. BMJ 2021;372:n71. doi: 10.1136/bmj.n71. This work is licensed under CC BY 4.0. To view a copy of this license, visit https://creativecommons.org/licenses/by/4.0/

Scale Analysis according to International Classification of Functioning (ICF)

Table aligned to ICF Chapters and Second-Level Categories with frequency of appearance and classification by domains:

- Body structures/functions: 18 scales
- Activity: 27 scales
- Participation: 17 scales
- Total of single scales: 62
- Total scales: 82

SCALE	FREQUENCY	ICF DOMAIN(S)	PRIMARY ICF CODE(S)	ADDITIONAL ICF CODE(S)	MAPPING NOTE
Epworth Sleepiness Scale (ESS)	1	Body functions	b134 Sleep functions	b1340,b1341,b1343	Daytime sleepiness reflects sleep amount/onset/quality
Pittsburgh Sleep Quality Index (PSQI)	1	Body functions	b134 Sleep functions	b1340,b1342,b1343,b1344	Sleep quality and maintenance
Visual Analogue Scale (VAS) – Pain	2	Body functions	b280 Sensation of pain	b2800-b2802	Pain intensity
Pain Disability Questionnaire (PDQ)	1	Body functions + Activities/Participation	b280 Sensation of pain	d230,d450	Pain impact on daily routine and mobility
Fatigue Severity Scale (FSS)	3	Body functions	b130 Energy and drive functions	b1300,b1301	Fatigue severity
Fatigue Impact Scale (FIS)	1	Body functions + Activities/Participation	b130 Energy and drive functions	d230,d850,d920	Fatigue impact on activities/participation
Modified Fatigue Impact Scale (MFIS)	1	Body functions + Activities/Participation	b130 Energy and drive functions	d230,d850,d920	Fatigue impact (physical,cognitive,psychosocial)
HADS (Anxiety/Depression)	2	Body functions	b152 Emotional functions		Affective symptoms
STAI (State–Trait Anxiety)	1	Body functions	b152 Emotional functions		Anxiety
RESE (Self-Efficacy)	1	Body functions	b126 Temperament and personality functions	b1301	Self-efficacy and motivation
MoCA	2	Body functions	b144 Memory functions; b140 Attention functions; b164 Higher-level cognitive functions	b168,b172	Global cognition
SDMT	3	Body functions	b140 Attention functions	b164	Processing speed/attention

PASAT (2s/3s)	2	Body functions	b140 Attention functions	b172 Calculation functions,b164	Working attention and mental arithmetic
ROCF (copy/recall variants)	1	Body functions	b156 Perceptual functions	b144 Memory,b1641 Organization and planning	Visuospatial construction and memory
SPART (10/36 Spatial Recall Test)	1	Body functions	b144 Memory functions	b1565 Visuospatial perception	Visuospatial learning/memory
SRT (CLTR / DR / LTS)	3	Body functions	b144 Memory functions		Verbal learning and memory
Word List Generation (WLG)	1	Body functions	b168 Mental functions of language	b164 Higher-level cognitive functions	Verbal fluency
BICAMS (SDMT + BVMT-R + CVLT-II)	1	Body functions	b140 Attention; b144 Memory	b156,b164,b168	Brief MS cognitive battery
CVLT-II	1	Body functions	b144 Memory functions	b168 Mental functions of language	Verbal learning/memory
BVMT-R	1	Body functions	b144 Memory functions	b1561 Visual perception	Visual learning/memory
Mini-BESTest	1	Activities	d415 Maintaining a body position	d450 Walking; b235 Vestibular; b760 Control of voluntary movement	Dynamic balance
Tinetti Balance and Gait	1	Activities	d415 Maintaining a body position; d450 Walking	b760	Balance and gait performance
Timed Up and Go (TUG)	1	Activities	d410 Changing basic body position; d450 Walking	d420 Transferring oneself; d455 Moving around	Functional mobility
T25-Foot Walk	1	Activities	d450 Walking	b455 Exercise tolerance functions	Walking speed
Timed Tandem Walk	1	Activities	d415 Maintaining a body position	d450 Walking	Tandem gait/balance
MSWS-12	2	Activities	d450 Walking	d455 Moving around	Walking limitations
NHPT (Nine-Hole Peg Test)	3	Activities	d440 Fine hand use	d445 Hand and arm use; b760	Manual dexterity
Box and Blocks Test (BBT)	1	Activities	d440 Fine hand use	d445 Hand and arm use	Gross manual dexterity
Grip strength (Jamar)	1	Body functions	b730 Muscle power functions	d430 Lifting and carrying objects	Maximal grip strength
Prone bridge test	1	Body functions + Activities	b740 Muscle endurance functions	d415 Maintaining a body position	Core endurance

Side-bridge test	1	Body functions + Activities	b740 Muscle endurance functions	d415 Maintaining a body position	Lateral core endurance
Trunk flexor test	1	Body functions + Activities	b740 Muscle endurance functions	d415 Maintaining a body position	Trunk endurance
Biering–Sorensen test	1	Body functions + Activities	b740 Muscle endurance functions	d415 Maintaining a body position	Back extensor endurance
Control Trunk Test	1	Activities	d415 Maintaining a body position	b760 Control of voluntary movement	Trunk control
Berg Balance Scale (BBS)	1	Activities	d415 Maintaining a body position	d450 Walking	Static and dynamic balance
2-Minute Walk Test (2MWT)	1	Activities	d450 Walking	b455 Exercise tolerance functions	Walking endurance
6-Minute Walk Test (6MWT)	2	Activities	d450 Walking	b455 Exercise tolerance functions	Walking endurance
G-Walk / wearable sensor gait analysis	1	Activities/Participation	d450 Walking; d455 Moving around	b770 Gait pattern functions	Instrumented gait performance
ICIQ-SF	1	Body functions + Activities	b620 Urination functions	b630 Sensations associated with urinary functions; d530 Toileting	Incontinence symptoms and impact
ICIQ-UI-SF	2	Body functions + Activities	b6202 Urinary continence	b6201 Frequency of urination; d530 Toileting	Urinary incontinence severity
ICIQ-UI-SF-QoL	1	Participation	b620 Urination functions	d530 Toileting; d920 Recreation and leisure	QoL impact of incontinence
OAB-V8	2	Body functions	b6201 Frequency of urination	b630 Urinary sensations	Overactive bladder symptoms
Leak number register	1	Body functions	b6202 Urinary continence	d530 Toileting	Leak episodes count
Voiding diary	1	Body functions	b620 Urination functions	b6201,b630; d530	Frequency/urgency episodes
Use of compresses/pads	1	Activities	d530 Toileting	d570 Looking after one’s health	Management strategy
EuroQol EQ-5D	1	Participation + Activities + Body functions	d450 Walking; d510–d540 Self-care	d230 Usual activities; b280 Pain; b152 Emotional functions	Generic health status
HAQUAMS	1	Participation + Activities + Body functions	d450; d510–d540; d230	b130 Fatigue; b152 Emotion; b280 Pain	MS QoL

		Participation + Activities + Body functions			
MSQoL-54	6	Participation + Activities + Body functions	d450; d510–d540; d230	b130; b152; b280	MS QoL
MusiQoL	1	Participation + Activities + Body functions	d450; d510–d540; d230	b130; b152; b280	MS QoL
QoLS	1	Participation	d230 Carrying out daily routine	d920 Recreation and leisure; d750 Informal social relationships	Global QoL
NHP-I	1	Participation + Body functions	d230 Carrying out daily routine	b280; b152; d450	Health perception
MSIS-29	1	Participation + Body functions	d4 Mobility; b152 Emotional functions	b130 Fatigue; b280 Pain	MS impact (physical + psychological)
MSISQ-19	1	Body functions + Activities	b640 Sexual functions	b670 Sensations assoc. with genital functions; d770 Intimate relationships	Sexual dysfunction in MS
MS-TAQ (treatment adherence)	1	Activities/Participation	d570 Looking after one's health		Adherence behaviours
Godin Leisure-Time Exercise Questionnaire	1	Participation	d920 Recreation and leisure	d570 Looking after one's health	Habitual physical activity
Sensor system (activity monitor)	1	Participation + Activities	d450 Walking; d455 Moving around	d920 Recreation and leisure	Real-world mobility
JAMAR CRT (Choice Reaction Time)	1	Body functions	b148 Psychomotor functions	b1480 Psychomotor control; b760 Control of voluntary movement	Response speed/psychomotor control
KVIQ (Kinesthetic and Visual Imagery Questionnaire)	1	Body functions	b156 Perceptual functions	b260 Proprioceptive function; b176 Sequencing complex movements	Imagery vividness (visual + kinesthetic)
Mental chronometry (imagery timing)	1	Body functions	b160 Pace of thought	b176 Sequencing complex movements	Imagery–execution temporal coupling

Complementary material 2. Quality of life scales. Additional psychometric information about the measures

QOL SCALE	DESCRIPTION	SUBSCALES	CUT-OFFS	MCID
Multiple Sclerosis Quality of Life (MSQoL-54)	MS-specific HRQoL measure combining SF-36 with MS modules<br>54 items<br>12 multi-item scales + 2 single ítems<br>Yields Physical and Mental composite scores, 0–100 (higher = better).	Physical health<br>Role limitations-physical<br>Role limitations-emotional<br>Pain<br>Emotional well-being<br>Energy<br>Health perceptions<br>Social function<br>Cognitive function<br>Health distress<br>Sexual function<br>Change in health<br>Satisfaction with sexual function<br>Overall QoL<br>Physical health composite<br>Mental health composite	No validated cut-offs.<br>Interpretation by score changes	Not established in literature
Multiple Sclerosis Impact Scale (MSIS-29)	Patient-reported measure of physical and psychological impact of MS<br>Raw domain sums transform to 0–100 (higher = worse impact)	Physical (20 items)<br>Psychological (9 items)	No validated severity cut-offs	Not established in literature
Kings Health Questionnaire (KHQ)	Disease-specific QoL instrument for urinary incontinence/overactive bladder.	General health<br>Incontinence impact	No universal cut-off categories are established.	5-10 point change in domain scores.<br>10 total score points is often

Complementary material 2. Quality of life scales. Additional psychometric information about the measures

	Domain scores 0–100 (higher = worse);	Role limitations<br>Physical limitations<br>Social limitations<br>Personal relationships<br>Emotions<br>Sleep/Energy<br>Severity		used as clinically meaningful
Hamburg Quality of Life Questionnaire Multiple Sclerosis (HAQUAMS)	MS-specific QoL measure addressing physical, cognitive, emotional, and social domains.<br>Rated 1–5 (higher = worse QoL)	Mobility<br>Fatigue<br>Cognition<br>Social function<br>Emotional well-being	No validated cut-offs	Not established in literature
Quality of life Scale (QoLS)	Generic QoL instrument assessing overall life satisfaction across multiple life domains.<br>Items 1–7, summed to a total score (higher = better)	Health/Functioning<br>Social/Economics<br>Psychological/Spiritual<br>Family<br>(Varies by versions)	No validated cut-offs	No universally accepted MCID established
Euro Quality of Life (EuroQoL)	Generic health-related QoL measure	Mobility<br>Self-care<br>Usual activities<br>Pain/Discomfort<br>Anxiety/Depression	No validated cut-offs are established	0.03–0.05 change in the utility index is considered minimally important only
Multiple Sclerosis Quality of Life (MusiQoL)	International MS-specific QoL instrument<br>Domain and global scores range 0-10 (higher=better QoL)	Activities of daily living<br>Psychological well-being<br>Symptoms	No validated cut-off points	No universally accepted MCID established

Complementary material 2. Quality of life scales. Additional psychometric information about the measures

		Relationships (friends/family/health care system)<br>Sentimental/sexual life<br>Coping<br>Symptoms<br>Rejection		
International Consultation of Incontinence Questionnaire-Short Form-Quality of Life (ICIQ-UI-SF-QoL)	Brief questionnaire assessing frequency, severity, and QoL impact of urinary incontinence<br>Scores range 0-21 (higher=worse)	Single total score plus individual items	Slight (1–5) Moderate (6–12, Severe (13–18, very severe (19–21)	Anchor-based estimates suggest a 4-point change is clinically important

Source: https://www.sralab.org/rehabilitation-measures
